# Case report: Individualized 3D-printed uncemented distal fibular prosthesis preserving the lateral malleolus for repair of distal fibular defects

**DOI:** 10.3389/fonc.2024.1380508

**Published:** 2024-08-29

**Authors:** Mengzhang Xie, Taojun Gong, Yitian Wang, Zhuangzhuang Li, Yuqi Zhang, Minxun Lu, Yi Luo, Li Min, Chongqi Tu, Yong Zhou

**Affiliations:** ^1^ Department of Orthopedics, Orthopedic Research Institute, West China Hospital, Sichuan University, Chengdu, China; ^2^ Model Worker and Craftsman Talent Innovation Workshop of Sichuan Province, West China Hospital, Sichuan University, Chengdu, China

**Keywords:** 3D-printed prosthesis, distal fibula, reconstruction, surgery, anlotinib, alveolar soft-part sarcoma

## Abstract

**Background:**

Involvement of the distal fibula by alveolar soft-part sarcoma is rare. Extensive resection or amputation may be needed; however, distal fibula resection can disrupt foot and ankle biomechanics, leading to ankle joint instability. Reports on joint preservation for maintaining optimal ankle joint function are scarce. Computer-aided design and individualized three-dimensional (3D)-printed uncemented implants represent an evolving solution for reconstructing the distal fibula.

**Case presentation:**

A 34-year-old woman was diagnosed with alveolar soft-part sarcoma in the right lower leg involving the cortical bone of the fibula. After anlotinib treatment, the tumor size decreased, and the tumor response rate was a partial response (PR); however, the patient continued to experience adverse reactions. With multiple disciplinary team discussions, surgical resection was deemed appropriate. Due to the extensive defect and ankle joint instability after resection, a custom-made 3D−printed prosthesis was designed and fabricated to reconstruct the defect, preserving the lateral malleolus. During the follow-up, the patient achieved favorable ankle function, and no prosthesis-related complications were observed.

**Conclusion:**

3D-printed personalized uncemented implants constitute a novel approach and method for addressing the reconstruction issues of the distal fibula and ankle joint. Through the personalized design of 3D-printed prostheses, the lateral malleolus can be preserved, ensuring the normal anatomical structure of the ankle joint. They achieve a well-integrated interface between the prosthesis and bone, ensuring satisfactory postoperative function. Additionally, they offer valuable insights for reconstructing distal bone defects near joints in the extremities. However, confirming these findings requires extensive cohort studies.

## 1 Introduction

Alveolar soft-part sarcoma (ASPS) is rare and represents less than 1.0% of soft tissue sarcomas (STSs) (1). ASPS mainly affects patients aged 15–35 years and occurs in deep soft tissues. There were more primary sites in the lower limbs than in the upper limbs (2). Bone involvement is rare (3). ASPS is resistant to chemotherapy. Surgical resection and/or systemic treatment for metastatic disease, immune checkpoint inhibitors (ICIs), and tyrosine kinase inhibitors (TKIs) are used to treat ASPS (2).

We report a 34-year-old woman who complained of swelling with an “egg” mass in the right lower leg for 1 month after exercise. Clinical examination revealed a 3.1-cm × 5-cm × 2-cm soft, raised, well-circumscribed, nontender mass located on the dorsal side of the right lower leg, and the pulses were normal in both dorsalis pedis arteries. A needle biopsy confirmed ASPS at Nanchong Central Hospital.

We present a patient with a biopsy-proven ASPS in the right lower leg. The patient initially underwent oral anlotinib-targeted therapy. After 12 cycles, the tumor exhibited reduction, although it remained present in the middle and lower tibia as well as a portion of the tibia. For more aggressive tumors, surgical resection or even amputation is often considered. Ankle biomechanics provide acceptable functional results (4) However, resection of the distal fibula can alter foot and ankle biomechanics, resulting in ankle joint instability. To preserve ankle function, reconstruction is generally recommended. After distal fibula resection, the approaches used for reconstruction include allograft reconstruction (5), arthrodesis (6), autografting (7, 8), and prosthesis implantation (9, 10).

In distal fibular resections without reconstruction, despite soft tissue reinforcement, ankle stability is not guaranteed (11), and proximal nonvascularized or vascularized fibular transposition and observation of the survival of the bone flap are difficult (12). Additionally, it may alter the knee’s stability (13) or endanger the peroneal external nerve (7, 14). While many of these methods can achieve satisfactory functional outcomes, they are often associated with complications and may not always be cost-effective. In this patient, ankle preservation surgery involved the utilization of a three-dimensional (3D)-printed porous prosthesis following *en bloc* resection of the distal fibula. 3D-printed uncemented implants are also a viable option for reconstructing bone defects. In comparison with allograft reconstruction in the distal radius, better wrist function was observed in early and midterm follow-ups with 3D-printed implants (15, 16). Similarly, acceptable early postoperative function has been reported with 3D-printed implants in pelvic bone tumor resections and the reconstruction of critical femoral bone defects (17, 18). This article reports a case of reconstructing a distal fibula defect using a 3D-printed implant, while preserving the external malleolus.

## 2 Manuscript formation

### 2.1 Case presentation

A 34-year-old woman was referred to our outpatient clinic in April 2021. Clinical examination revealed a 3.1-cm × 5-cm × 2-cm soft, raised, well-circumscribed, nontender mass located on the dorsal side of the right lower leg, and the pulses were normal in both dorsalis pedis arteries. A needle biopsy confirmed ASPS at Nanchong Central Hospital. No metastasis to other sites was found. Heart and lung function tests revealed no obvious abnormalities.

**Figure 1:**
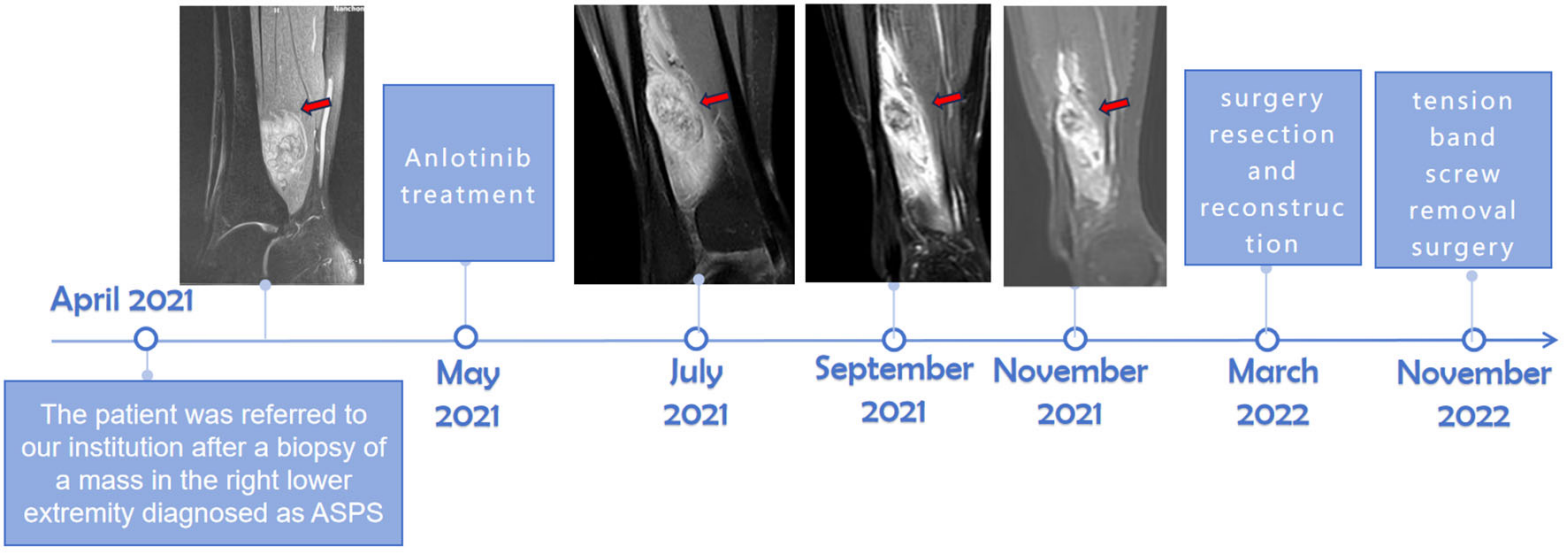
The timeline of the patient’s diagnosis and treatment. Magnetic resonance imaging (MRI) revealed a substantial extraskeletal tumor surrounding the fibula, as indicated by the arrow, prior to anlotinib. Subsequently, the size of the tumor decreased after anlotinib treatment.

Between May and November 2021, the patient received six courses of anlotinib treatment (12 mg once daily; 2 weeks on/1 week off, and the treatment cycle lasted 3 weeks). The patient reported that the mass decreased, and the tenderness disappeared. Magnetic resonance imaging (MRI) was performed once every 3 months, and the size of the tumor ranged from 3.3 cm × 2.9 cm to 2.3 cm × 1.7 cm. According to the Response Evaluation Criteria for Solid Tumors (RECIST) 1.1, the efficacy of anlotinib is classified as a partial response (PR). However, MRI revealed disease progression in the right distal fibula from September to November, and the tumor involved the bones by extending the soft tissue tumor. The patient experienced adverse reactions, including hand–foot skin reaction (HFSR) and gastrointestinal adverse reactions. After multiple interdisciplinary team (MDT) discussions and in accordance with the patient’s wishes, surgery was performed in March 2022 (details are provided in **Figure 1**).

### 2.2 3D-printed fibula prosthesis design and manufacture

**Figure 2:**
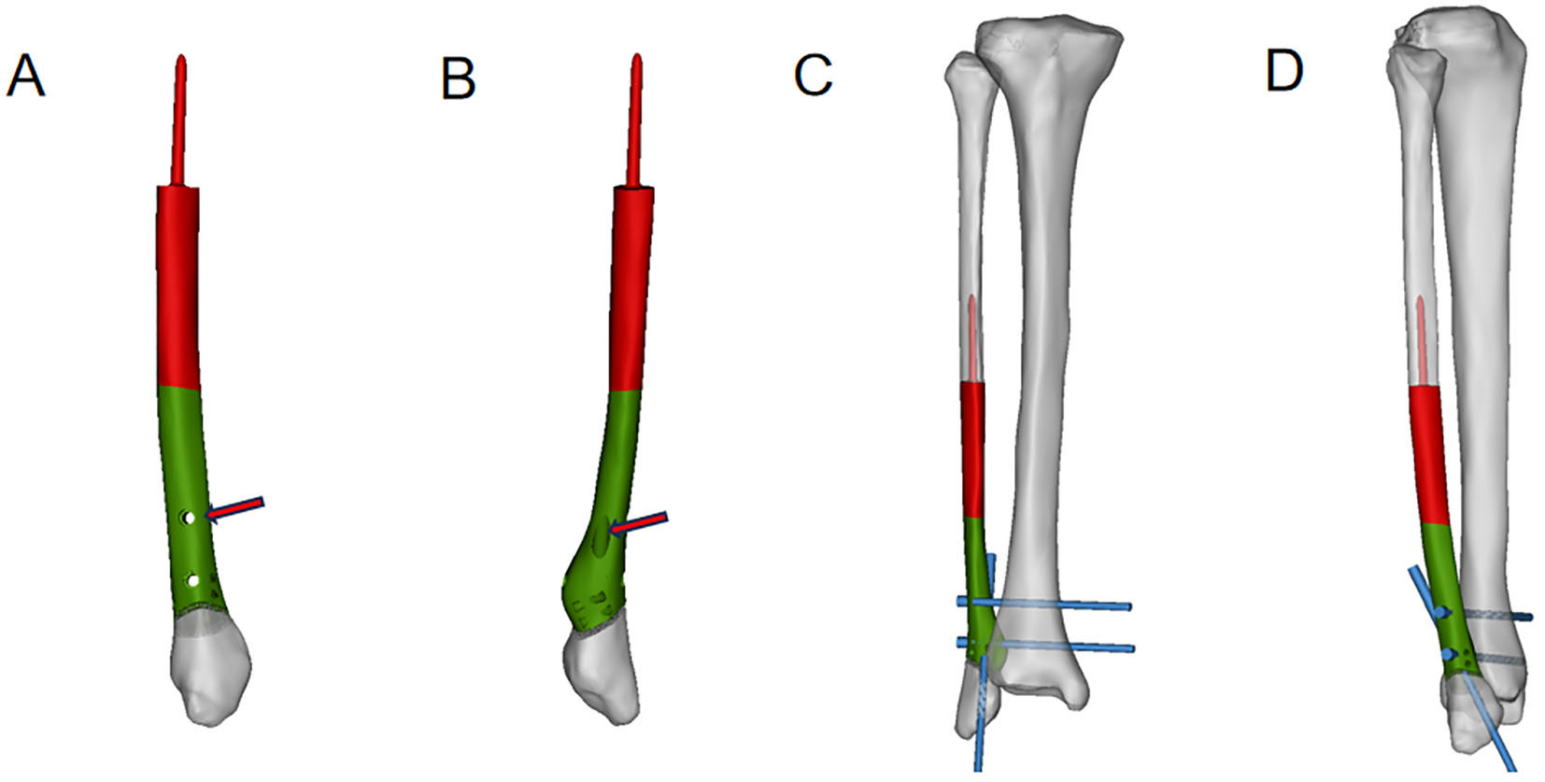
Products of 3D−printed prostheses.

The 3D-printed prosthesis in this case was modeled by our team and produced by Beijing Chunlizhengda Medical Instruments Co. (Chun-Li, Beijing, People’s Republic of China). First, the CT data of both lower limbs of the patient were imported into Mimics software (Materialise Corp., Leuven, Belgium) to determine the resection range of the fibula and to retain the lateral malleolus, and a preliminary prosthesis model was established (S1). Considering that the distal resection surface of the fibula is inclined and that some cortical tumors invading the posterior part of the distal tibia need to be scraped away, the connection between the distal end of the prosthesis and the reserved lateral malleolus was designed as a porous structure with a pore diameter of 600 nm and a porosity of 70%. Two screws were designed at the distal end of the fibula to connect the tibia and fibula, and one screw was designed to connect the prosthesis to the remaining distal fibula to maintain the stability of the prosthesis and bone (**Figure 2**). Meanwhile, suture holes were reserved at the distal end of the prosthesis and the distal end of the residual fibula to strengthen the integration of the prosthesis and bone. The main components of the 3D-printed prosthesis include a titanium alloy rod and a stem coated with hydroxyapatite and titanium powder that was plasma sprayed, which is inserted into the proximal medullary cavity of the fibula (S2). The prosthesis was printed using electron beam melting technology (ARCAM Q10, Mölndal, Sweden).

### 2.3 Surgical techniques

**Figure 3:**
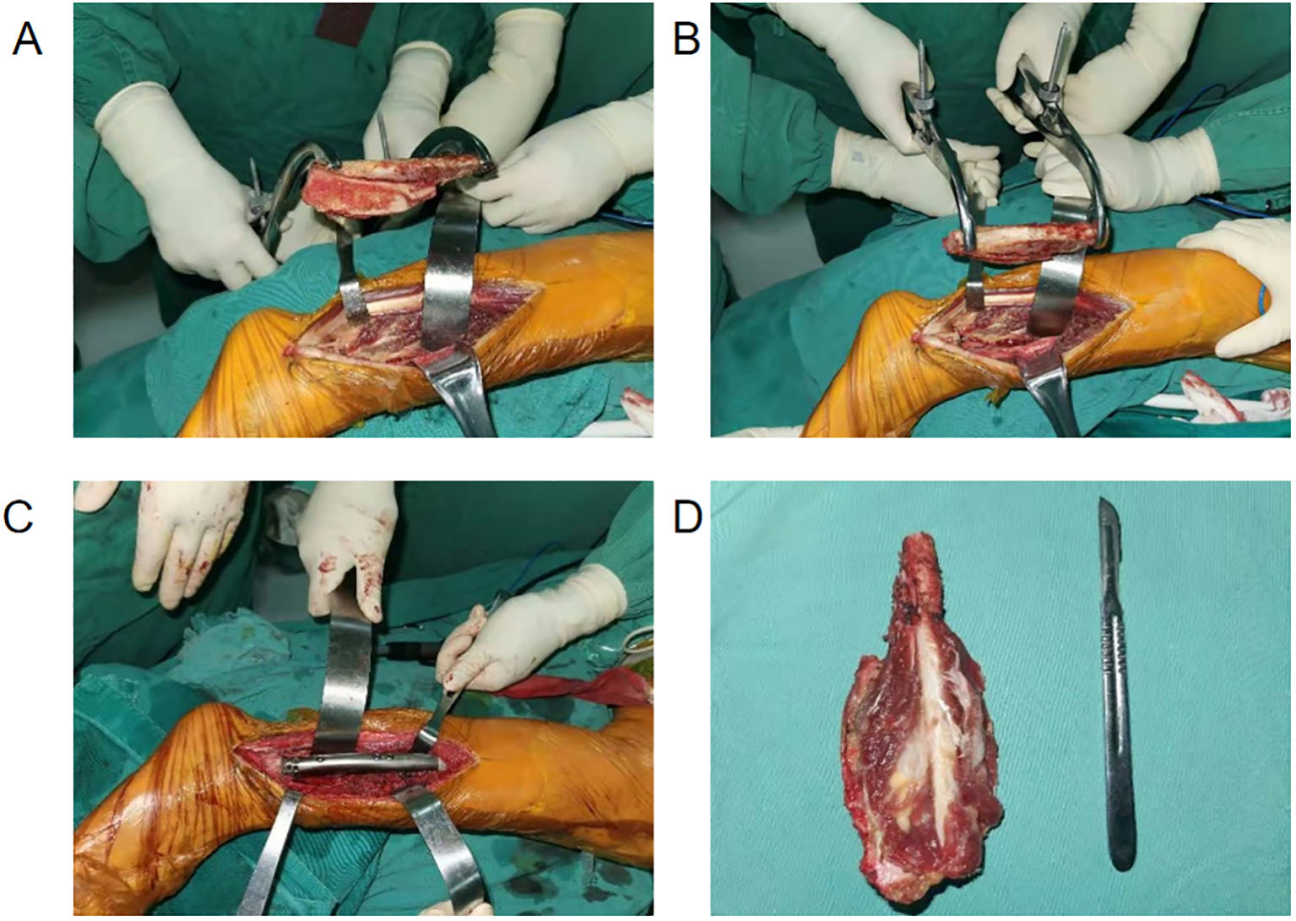
Intraoperative images indicate the resection of the middle and distal fibula via a lateral approach. **(A, B)** The resected tumor and distal fibula in different directions were observed externally. **(C)** Implantation of the prosthesis was completed. **(D)** Dissection of the tumor segment revealed that the tumor tissue was gray–yellow, gray, and white with soft fish-like stroma.

The surgery was performed by a senior surgeon. The patient was placed in a left-lateral position with a lateral approach. During the procedure, most of the involved flexor hallucis longus, tibialis posterior, and soleus muscles were resected. A vernier caliper was used to measure and mark the osteotomy surface of the fibula (distal to the tip: internal, 2.5 cm; external, 3.0 cm; proximal to the distal external osteotomy surface approximately, 11 cm). Osteotomy was carried out with a micropendulum saw directly to the external and posterior sides of the tibia along the fibula, and the cortex behind the affected fibula outside the tumor was directly removed (**Figures 3A, B**). The proximal part of the prosthesis was implanted into the fibular bone marrow cavity, and then the distal part of the prosthesis was fixed with 3.0 mm diameter screws and sutures, while the prosthesis and fibula were fixed with two 3.5 mm diameter screws (**Figure 3C**). The cancellous bone allograft was implanted into the tibial defect and the distal tibiofibular defect. The size of the tumor was approximately 6 cm × 5 cm × 3 cm, and it involved approximately 3 cm of the posterior cortex of the tibia, approximately 6 cm of the medial posterior part of the fibula, and 1 cm above the malleolus of the posterior inferior tibiofibular ligament (**Figure 3D**).

### 2.4 Results

**Figure 4:**
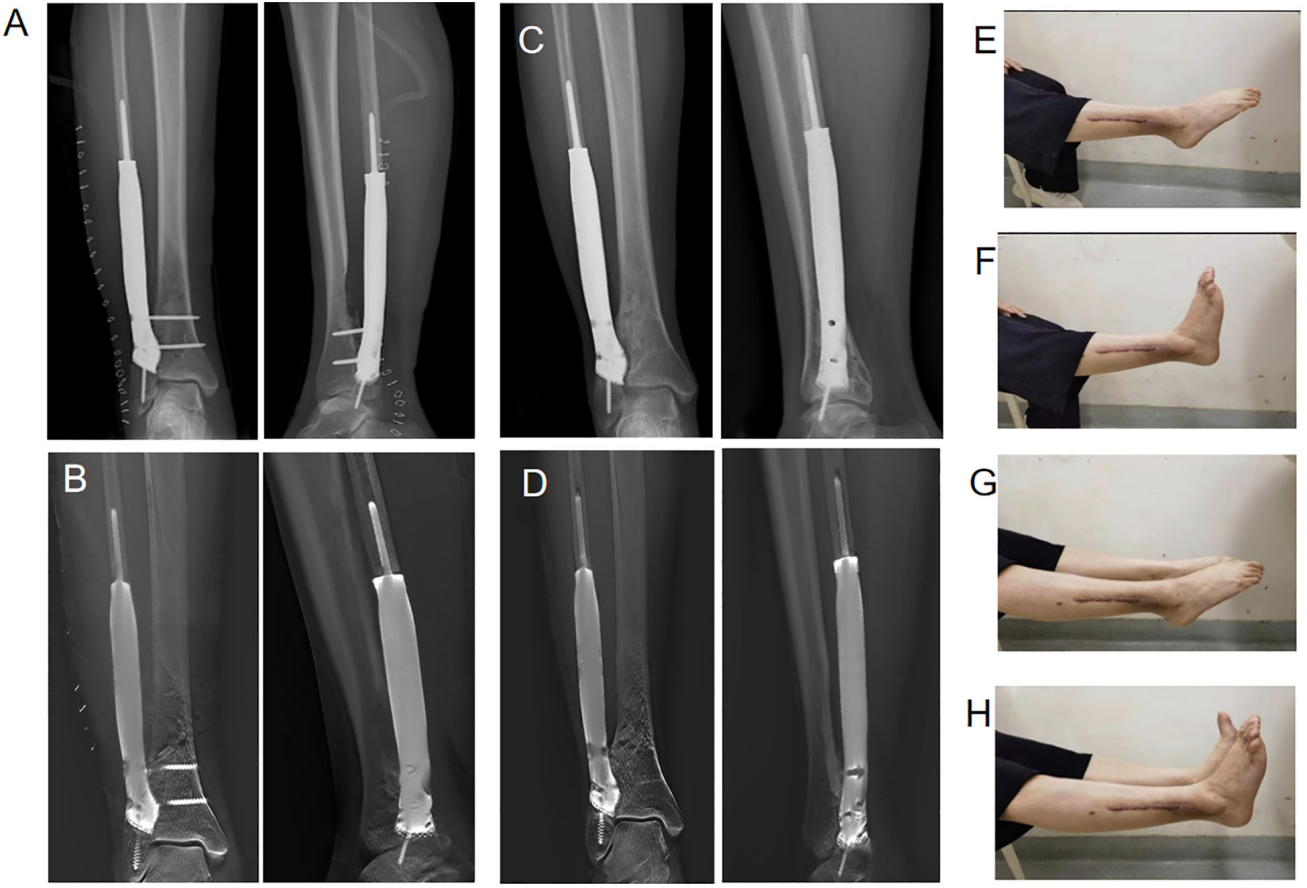
Postoperative imaging at 3 and 21 months and activity involving plantar flexion and dorsiflexion of the right ankle joint. **(A)** Postsurgical anteroposterior and lateral radiographs at 3 months. **(B)** Postsurgical anteroposterior and digital tomosynthesis images at 3 months. **(C)** Postsurgical anteroposterior and lateral radiographs at 21 months. **(D)** Following the extraction of screws from the prosthesis and tibia, anteroposterior and lateral postsurgical digital tomosynthesis revealed that the prosthesis was tightly integrated with the distal fibula, and partial bone substance was observed to grow into the pores of the prosthesis. **(E, F)** Activity involving plantar flexion and dorsiflexion of the right ankle joint. **(G, H)** Simultaneous motion of both ankles indicated that the range of motion in the ankle joint on the affected side was nearly identical to that on the healthy side.

Functional recovery of the ankle was satisfactory 3 months postsurgery. The patient underwent regular, monthly follow-up appointments. After evaluation, the tibiofibular lag screw was removed from the right tibial prosthesis at our hospital on 22 November 2022. At the last follow−up on 20 December 2023, postoperative imaging indicated integration of the 3D-printed prosthesis with both the proximal and distal fibula. Simultaneously, the anatomical structure of the distal fibula in the affected limb remained normal after the screws were removed. No complications, such as prosthesis displacement, aseptic breakage, or loosening, were observed (**Figures 4A–D**). The range of motion of the ankle was almost normal compared with that of the healthy side (**Figures 4E–H**). The functional scores were 97% for the MSTS−93 score, 95% for the AOFAS score, and 95% for the TESS score. During the follow−up, there was no wound infection, skin necrosis, or surgical-related complications. No cases of varus deformity or valgus deformity were found. The patient underwent follow-up lung CT scans and head-abdominal MRI scans after surgery. The most recent follow-up showed no evidence of tumor metastasis or recurrence.

### 2.5 Discussion

For ASPSs involving bones, local surgical control is critical (19). In this case, a 3D-printed prosthesis was used for the reconstruction of the distal fibula and the preservation of the lateral malleolus. Due to improvements in limb salvage surgery technology, the requirements for postoperative joint function are also increasing. The challenging aim of surgery is to promote the recovery of ankle joint function and reduce postoperative complications.

Our study had some unavoidable limitations. This study included one sample with short-term follow-up, and prosthesis-related complications may not have been present. The superiority of 3D-printed prostheses over other surgical techniques cannot be verified in one patient. There was no biomechanical analysis included in our study; more patients undergoing this procedure are needed to perform comparative analysis with other operations and finite element analysis to further optimize the prosthesis design. This study primarily describes the success of the implant through auxiliary examinations and patient function evaluations. However, a definitive determination can only be made by removing the implant.

Diverse techniques for reconstructing the distal fibula after wide tumor resection have been documented, each with distinct advantages and disadvantages. Ankle arthrodesis has the advantages of improved pain relief and a decreased revision rate (20). Dieckmann et al. (7) completed arthrodesis via a retrograde nail, which involves stable arthrodesis, intramedullary stabilization of the tibia, and avoidance of extrinsic material in the wound area. The most serious weakness of ankle arthrodesis is the sacrifice of tibiotalar joint motion (21).

Autografts mainly include vascularized fibula transposition and nonvascularized fibula transposition. Vascularized fibular transposition ensures that the blood supply for the epiphyseal plate promotes bone formation via the peroneal vessels (22). Therefore, the growth of the bone graft can be ensured, the ankle joint can be stabilized and preserved, and the contralateral limb can be normal. Rajacic (23) employed a vascularized autograft, resulting in improved postoperative function. The ankle joint remained stable, and normal growth was observed 4 years after surgery. However, as mentioned earlier, autografts have limitations, and the risk of failure or fracture in devitalized autografts poses a challenge because of the demanding nature of the procedure.

We report a case of distal fibular intercalary reconstruction using a 3D-printed prosthesis. During preoperative communication, the advantages and disadvantages of each surgical plan were thoroughly explained to the patient. Considering his age, the patient deemed ankle function very important. Our institution has extensive experience and numerous cases of 3D-printed prosthesis implantation. The patient also preferred the 3D-printed prosthesis for achieving complete anatomical reduction and was resistant to transplantation surgery. Consequently, we reached an agreement with the patient to proceed with this surgical plan. Reconstruction of the distal fibula while preserving the lateral malleolus joint is relatively rare. For distal fibular tumors, the required resection often precludes preserving the lateral malleolus joint. In this case, the length of the distal fibular osteotomy was 11 cm, with the distal end located 3.5 cm from the lateral malleolus, making it possible to preserve the normal anatomical structure of the ankle joint.

Although the fibula is often used as an autograft for intercalary reconstruction of long bone segmental defects, fibular defects generally do not require reconstruction. However, in this case, the remaining distal fibula was too short, necessitating reconstruction of the defect. Typically, distal fibular reconstructions do not preserve the lateral malleolus, but pedicled vascularized fibula transposition and intercalary allografts or their combined use can preserve the ankle joint (**Table 1**).

**Table 1:** The methods of distal fibula reconstruction.

Reconstruction type	Author	Number of cases	Median follow-up time (months)	Preservation of lateral malleolus preservation of lateral malleolus	Complications	Shortcomings
Ankle joint arthrodesis	Dieckmann (7)	9	40	No	Fracture, delayed wound healing, infection, pseudarthrosis with talipes equinus, intralesional resection	Part of the fusion needs a fibula graft or more screws to reinforce the ankle; sacrificing the tibiotalar joint motion
	Khal (4)	3	–	No		
	Wang (24)	3	56.4	No		
Pedicled vascularized fibula transposition	Khal (4)	1	–	Yes	Peroneal nerve palsy Chronic pain; subluxation of talus	In terms of anatomical structure, the lateral genicular joint was unstable. The incongruity of the fibular head with the talus of the joint, which produces subluxation of the talus, still leaves open the possibility of arthrodesis
	Dieckmann (7)	1	155	No		
	Rajacic (23)	1	48	No		
	de Gauzy (8)	1	30	No		
Nonvascularized fibula transposition	Khal (4)	2	–	No	Chronic pain; decreased ankle mobility	In terms of anatomical structure, the lateral genicular joint was unstable. The incongruity of the fibular head with the talus of the joint, which produces subluxation of the talus, still leaves open the possibility of arthrodesis
	Wang (24)	3	104.7	No		
	Karmakar (25)	1	12	No		
	Khal (4)	1	–	Yes		
3D-printed prosthesis	Cheng (10)	1	24	No	Screw breakage	The follow-up time is short, and the cost is high. The degree of postoperative recovery is closely related to the design of the prosthesis, so the guidance of experienced surgeons is needed

There is no clear guideline for the selection of the reconstruction method. Allograft availability is limited, and the quality of allografts can vary based on donor age, health status, and processing methods, affecting the success of the graft and potentially causing immune rejection in the recipient (26). Complications such as allograft fractures, infections, and nonunion are reported in femoral intercalary reconstructions (27). In tibial intercalary reconstructions, the 5-year complication-free survival rate is 58%, with early postoperative complications including common peroneal nerve palsy, fractures, and nonunion. Vascularized fibula transposition can serve as a salvage therapy in cases of complications (28). Despite short-term follow-up studies showing no significant complications with lateral malleolus-preserving methods, donor and graft site complications remain a concern up to 5 years postoperatively (4).

In this case, a 3D-printed prosthesis tailored to the patient’s anatomical structure was used for reconstruction. This approach ensures a match to the site of the defect, offers high patient acceptance, and better aligns with the biomechanical requirements of the lower limb while promoting better osseointegration and reducing implant weight while maintaining mechanical strength. It eliminates the need for graft harvesting, reducing surgical time and risks, and avoiding donor site complications. The prosthesis provides immediate mechanical stability. At 3 months postoperatively, digital tomosynthesis images demonstrated good bone healing at the interface without signs of bone resorption or prosthetic infection (**Figure 4B**), and the absence of mechanical complications within the first 3 months indicates successful integration of the prosthesis into the patien’s body.

In this study, we employed a prosthesis to reconstruct a bone defect in the fibula while preserving the autologous lateral malleolus, thus nearly preserving the ankle joint entirely. The two screws used to reinforce the connection between the distal end of the prosthesis and the fibula were removed after 8 months of follow-up. No obvious complications, such as prosthesis fracture or displacement, were observed after removal. This also demonstrates that the slope design of the distal part of the prosthesis can enhance the bone regeneration ability of the integrated interface.

We summarize the key points of using 3D prostheses to achieve better functional recovery and fewer postoperative complications. First, the increased prosthesis length addressed soft tissue tension to stabilize the implant. Second, two screws were passed through the contralateral bone cortex to enhance immediate stability. Additionally, a conical stem coated with titanium and hydroxyapatite could be firmly inserted into the proximal cavity to guarantee the stability of the fixed. Third, the solid internal structure contributed to preventing prosthesis fracture. Fourth, the design of the prosthesis and the distal peroneal interface incorporates a lattice structure. This design provides a large surface area, which facilitates bone cell adhesion and proliferation and allows for vascular ingrowth. This ensures the necessary supply of nutrients and oxygen. Additionally, the porous structure reduces the weight of the implant while maintaining sufficient mechanical strength through an optimized design. In addition to prosthesis design, appropriate surgical techniques, early and scheduled rehabilitation programs, ASPS treatment, and regular follow-up to exclude metastasis are also crucial for patient recovery.

A specific pore size and porosity can induce bone ingrowth and integration (29). Due to the advantages of 3D printing in the personalized design of prostheses, we were able to preserve the patient’s original lateral malleolar structure without compromising the patient’s own ankle joint. This is crucial for the recovery of ankle joint function. Compared to fibula grafting and ankle arthrodesis, 3D-printed prostheses retain the ligaments and anatomical structures of the ankle joint, thereby eliminating factors that contribute to ankle instability and restricted movement. However, reconstructing an ultracritical bone defect with a prosthesis is challenging and susceptible to mechanical complications (30). Cheng et al. (10) reported a patient with distal fibular and lateral malleolar defects treated with a 3D-printed prosthesis whose ankle joint appearance and function were nearly normal, with no valgus deformity. Postoperative complications (screw breakage) were noted. Mechanical complications, such as loosening of the prosthesis, are likely to occur.

### 2.6 Conclusion

The use of 3D-printed personalized uncemented implants is an alternative approach and method for addressing the reconstruction issues of the distal fibula and ankle joint. Through the personalized design of 3D-printed prostheses, the lateral malleolus can be preserved, ensuring the normal anatomical structure of the ankle joint. Lattice structure helps promote an interface between the prosthesis and bone, contributing to satisfactory postoperative function. Additionally, they offer valuable insights for reconstructing distal bone defects near joints in the extremities. However, confirming these findings requires extensive cohort studies.

## Data Availability

The original contributions presented in the study are included in the article/**Supplementary Material**. Further inquiries can be directed to the corresponding author.
