# Supplementary figures and images for: Case report: Individualized 3D-printed uncemented distal fibular prosthesis preserving the lateral malleolus for repair of distal fibular defects
Source: Front Oncol. 2024 Aug 29;14:1380508. doi: 10.3389/fonc.2024.1380508 (PMC11390384; doi:10.3389/fonc.2024.1380508)

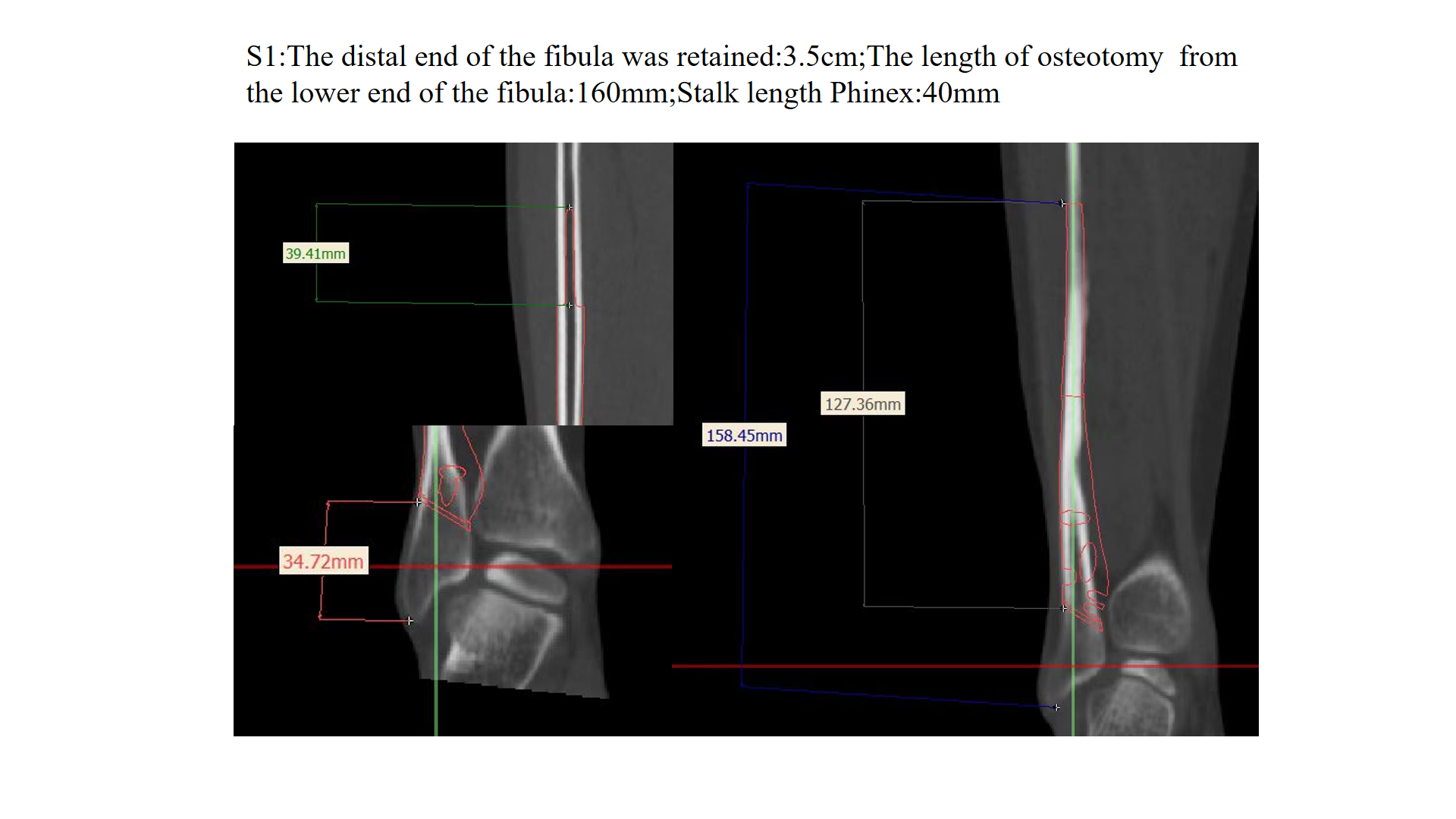

Supplement: Supplementary file 1 [file Image1.tif]

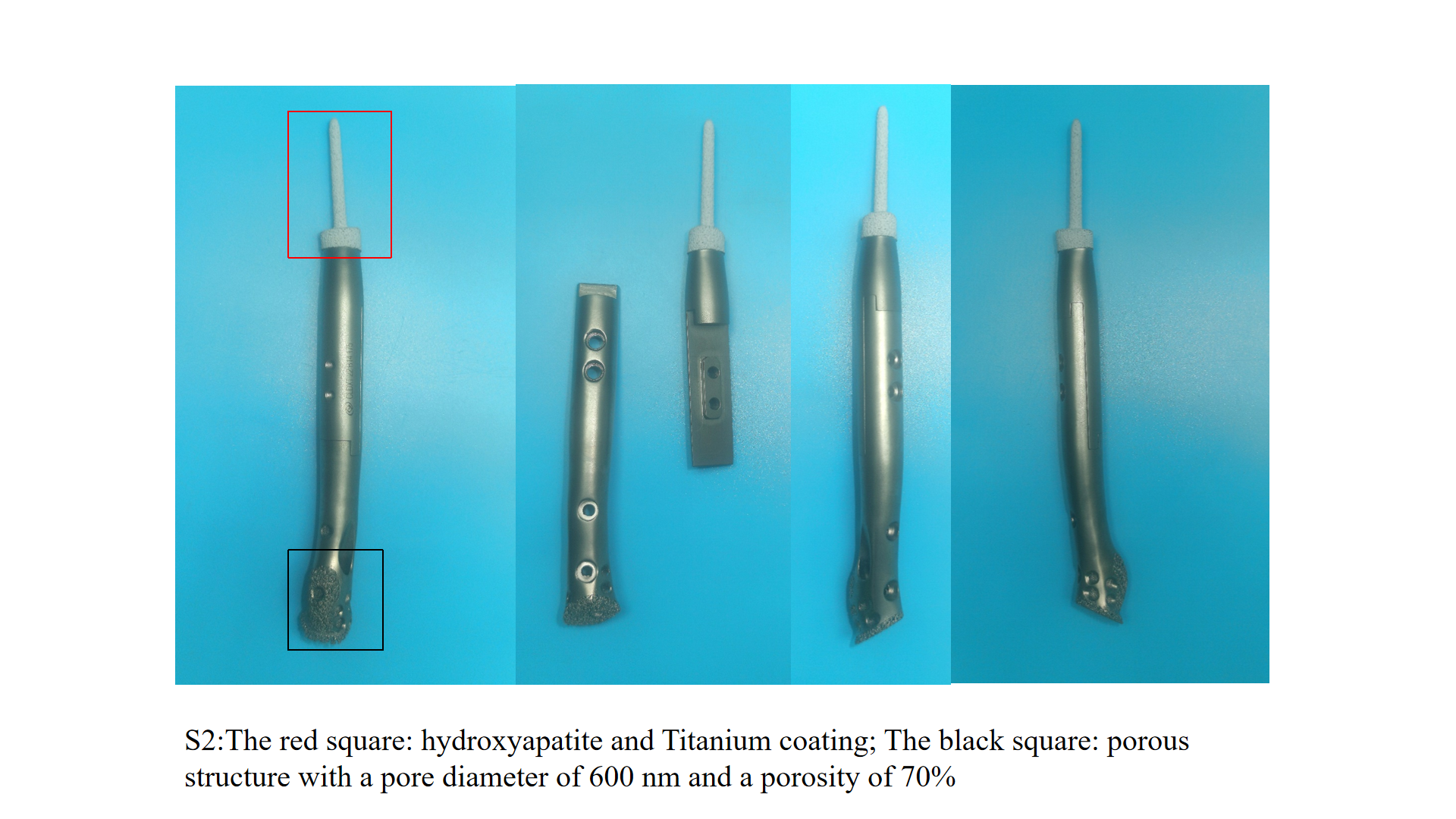

Supplement: Supplementary file 2 [file Image2.tif]
